# On-Going Frontal Alpha Rhythms Are Dominant in Passive State and Desynchronize in Active State in Adult Gray Mouse Lemurs

**DOI:** 10.1371/journal.pone.0143719

**Published:** 2015-11-30

**Authors:** Francesco Infarinato, Anisur Rahman, Claudio Del Percio, Yves Lamberty, Regis Bordet, Jill C. Richardson, Gianluigi Forloni, Wilhelmus Drinkenburg, Susanna Lopez, Fabienne Aujard, Claudio Babiloni, Fabien Pifferi, IMI project "PharmaCog" Consortium

**Affiliations:** 1 IRCCS San Raffaele Pisana, Rome, Italy; 2 UMR 7179 Centre National de la Recherche Scientifique, Muséum National d'Histoire Naturelle, Brunoy, France; 3 IRCCS SDN, Naples, Italy; 4 UCB Pharma s.a., Neuroscience Therapeutic Area, Braine l'Alleud, Belgium; 5 L'Université Lille 2 Droit et Santé, Lille, France; 6 Neurosciences Therapeutic Area Unit, GlaxoSmithKline R&D, Gunnels Wood Road, Stevenage, United Kingdom; 7 Department of Neurodegeneration, Mario Negri Institute, Milan, Italy; 8 Janssen Research & Development, Turnhoutsewe, Beerse, Belgium; 9 Department of Physiology and Pharmacology, University of Rome "La Sapienza", Rome, Italy; Universiteit Gent, BELGIUM

## Abstract

The gray mouse lemur (*Microcebus murinus*) is considered a useful primate model for translational research. In the framework of IMI PharmaCog project (Grant Agreement n°115009, www.pharmacog.org), we tested the hypothesis that spectral electroencephalographic (EEG) markers of motor and locomotor activity in gray mouse lemurs reflect typical movement-related desynchronization of alpha rhythms (about 8–12 Hz) in humans. To this aim, EEG (bipolar electrodes in frontal cortex) and electromyographic (EMG; bipolar electrodes sutured in neck muscles) data were recorded in 13 male adult (about 3 years) lemurs. Artifact-free EEG segments during active state (gross movements, exploratory movements or locomotor activity) and awake passive state (no sleep) were selected on the basis of instrumental measures of animal behavior, and were used as an input for EEG power density analysis. Results showed a clear peak of EEG power density at alpha range (7–9 Hz) during passive state. During active state, there was a reduction in alpha power density (8–12 Hz) and an increase of power density at slow frequencies (1–4 Hz). Relative EMG activity was related to EEG power density at 2–4 Hz (positive correlation) and at 8–12 Hz (negative correlation). These results suggest for the first time that the primate gray mouse lemurs and humans may share basic neurophysiologic mechanisms of synchronization of frontal alpha rhythms in awake passive state and their desynchronization during motor and locomotor activity. These EEG markers may be an ideal experimental model for translational basic (motor science) and applied (pharmacological and non-pharmacological interventions) research in Neurophysiology.

## Introduction

Gray mouse lemur (*Microcebus murinus*) is a nocturnal prosimian primate originating from Madagascar, which shares some genetic, physiological, and neuroanatomical similarities with humans. Marked biological rhythms have been extensively studied in the captive colony of mouse lemurs at CNRS/MNHN in Brunoy [1, 2, 3]. Its longevity (life expectancy of about 8 years) is ideal to carry out longitudinal studies on the effects of aging [4, 5]. Behavioral activity decreases at about 5 years of age [6, 7], in a framework of a global decline of executive functions, memory, and daily locomotor activity. Furthermore, cerebral atrophy was observed in both physiological and pathological aging [8]. Moreover, Aβ1–42 immuno-positive plaques, which are one of the pathophysiological hallmarks of Alzheimer's disease (AD), were found in the brain of some lemurs as early as 4 years of age [9]. Mouse lemur has, therefore, been proposed as one of the most interesting animal models for the translational study of AD in non-human primates [4]. It is expected that this animal model will provide effective biomarkers to monitor brain functions and neurodegenerative processes with aging. Furthermore, these biomarkers may be useful for preclinical investigations on the efficacy of new drugs for patients with neurodegenerative diseases [4, 5, 8].

Several neuroimaging methods allow the translational exploration of brain functions across species including mouse lemurs. Optical fluorescence imaging monitors biological functions of specific targets in small animals, but the intrinsic fluorescence of biomolecules poses some methodological problems [10, 11]. Task-independent functional brain neuroimaging by magnetic resonance imaging (MRI) allows examination of the intrinsic networks within the brain not only in humans but also in awake animals [12, 13, 14]. These networks can be defined as functionally and (often) structurally connected populations of neurons whose properties reflect fundamental neurobiological organizational principles of the central nervous system [14]. Neuroimaging techniques have considerable merits but a remarkable limitation for the study of brain function in animal models. They require that animals be constrained in limited spaces and unusual conditions of housing during the exam [12, 13, 14]. This methodological limitation does not apply to electroencephalographic (EEG) recordings. These recordings can be performed in freely behaving animals for extended periods of time, thus allowing the measurement of brain activity in the rest and activity phases of the wake-sleep cycle [2, 15, 16]. Among EEG techniques, those using telemetric wireless devices represent a very attractive methodology, in order to reduce the stress in the animal and make fully free its movements in the cage [16]. For this reason, EEG research may play a fundamental role in the study of characteristic biomarkers in wild-type and transgenic animal models of several diseases in the framework of a genuine back-translation model of research [17, 18, 19].

As another significant merit, EEG recording can probe the so-called synchronization and desynchronization of brain neural activity across wake-sleep cycles, as a function of pharmacological and non-pharmacological interventions [19, 20]. The synchronous oscillating activity of brain neural networks or its desynchronization result from the interaction among many neurons by their functional connectivity [21, 22]. Such synchronization and desynchronization of brain neural activity couple or decouple neuronal populations in several parallel cortical and sub-cortical networks. This neurophysiological mechanism generates and modulates the so-called cortical EEG rhythms, and is considered as an important neural substrate of vigilance, spatial orientation, consciousness, and planning of the behavior [23, 24, 25].

Are cortical EEG rhythms a useful translational model of the mentioned neurophysiological mechanisms in human and sub-human primates? In humans, resting state eyes-closed EEG oscillations at 8–12 Hz are dominant in posterior sensory (i.e. 8–10 Hz, alpha rhythms) and frontal motor areas (i.e. 10–12 Hz, Rolandic alpha or mu rhythms), as a reflection of cortical inhibition and behavioral quiet wakefulness [26]. Alpha rhythms are generated by a complex pattern of parallel cortico-cortical and cortical-subcortical (thalamic)-cortical neural networks [26]. These networks regulate the fluctuation of cortical arousal, tonic alertness, and muscle relaxation [26]. When an individual is engaged in some cognitive (i.e. attention, working memory) and sensorimotor events, parietal-frontal alpha rhythms disappear, the so-called de-synchronization or blocking of alpha rhythms [26]. These rhythms are replaced by parietal-frontal fast EEG oscillations at frequencies higher than 8–12 Hz such as beta (about 20–30 Hz) and gamma (>30 Hz) [26, 27, 28, 29]. In this vein, parietal-frontal alpha rhythms desynchronized in monkeys during cognitive-motor tasks [30].

In the IMI PharmaCog project (www.pharmacog.org), we have tested the translational value of spectral EEG markers in lemurs towards future translational applications to diseases that are apparent with aging, such as AD. In this framework, the present study tested the hypothesis that EEG rhythms in premotor frontal cortex accompanying motor and locomotor activity in prosimian lemurs showed similarities with respect to those observed in monkeys and humans. Specifically, we predicted ample alpha rhythms in the premotor frontal cortex during relaxed wakefulness (i.e. passive state) and their desynchronization during motor and locomotor activity (i.e. active state). We focused on premotor cortex as it has large domains for ethologically relevant movements such reaching, grasping, defensive, and other complex movement patterns in primates [31]. This function depends on the abundant signals coming from prefrontal cortex, which allows the coupling of information processing on cognitive (i.e. plans, environmental stimuli) and motor events [31, 32]. Its cognitive-motor function is of extreme interest towards future translational studies on aging and cognitive decline. Compared with premotor cortex, the primary motor cortex of primates shows more limited functions in the executive generation of voluntary hand and digit movements on objects and intra-personal body parts [31]. This function depends on the abundant signals coming from the motor thalamus, which receives signals from the cerebellum and basal ganglia [31]. In the present study, on-going EEG rhythms were recorded from premotor frontal cortex during passive and active behavioral states. Results confirmed the working hypothesis.

## Methods

### Animals and surgery for implantation of telemetric sensors

In the present study, we utilized thirteen male gray mouse lemurs (*Microcebus murinus*), which were born and raised in the laboratory breeding colony of Brunoy, France (agreement C-91-564). The original stock come more than 40 years ago from the southwest coast of Madagascar. All animals were disease free. The general conditions of captivity were maintained with respect to ambient temperature (25°C) and relative humidity (55%) during all the experiments. Individual cages (40cm x 40cm x 35cm) were provided with wooden nest box (at least two per animal) and fresh laurel branches for the whole duration of the experiment. The lemurs were adult (mean age 3.2 years ±0.5 standard error, SE), cognitively intact, and underwent no drug manipulation, sleep deprivation or other challenges or interventions before or in the period of the present EEG recordings.

All experiments were performed in accordance with the “Principles of Laboratory Animal Care” (National Institutes of Health publication 86–23, revised 1985) and the European Communities Council Directive (86/609/EEC). The research was conducted under the authorization number 91–305 from the ‘‘Direction Départementale de la Protection des Populations”. The experimental protocol was approved by the Cuvier Ethics Committee under the agreement number 068–018. Noteworthy, this protocol was specific for the study reported in this paper (EEG recordings in gray mouse lemurs were performed in the context of the IMI European project entitled “Pharmacog”). In accordance with international guidelines, particular attention was paid to the welfare of the animals during this work to minimize nociception (Weatherall FRS D. 2006. “The use of non-human primates in research. The Weatherall report”). No animal was sacrificed for the purpose of this study.

Surgeries were performed by the UMR CNRS-MNHN staff under the supervision of Dr. Fabienne Aujard (PhD, Veterinarian with the authorization to experiment in live animals number C-91-564 delivered by the French “Direction Départementale de la Protection des Populations de l'Essonne”). Experiments were conducted in an official experimental facility under the agreement number D-91.114.1 delivered by the French “Direction Départementale de la Protection des Populations de l'Essonne”.

Experimental plan of the present experiments included the use of a wireless telemetry system to collect physiological data such as EEG, electromyographic (EMG), temperature, and locomotor activity (Data Science International, St. Paul, MN, USA). To do so, a small transmitter with a volume of 1.9 cc and weighing 3.9 g (model PhysioTel F20-EET, DataScience Co. Ltd, Minnesota, USA) was implanted into the peritoneal cavity of the lemurs under a ketamine anaesthesia (Imalgene, 100 mg/kg intraperitoneal) empowered by administration of Metacam (meloxicam, 0.2 mg/kg).

Silicone elastomer insulated stainless-steel electrode wires (Ø 0.3 mm) were then subcutaneously led, passing between the scapula, from the abdomen to the skull, and there sealed using dental cement.

The EEG implanted electrodes were located in premotor cortex (Brodmann area 6, BA 6) in the anterior position of 7.00 mm according to the transversal section, which corresponded to the plate 34 of the "Stereotaxic atlas of the brain grey mouse lemur" [33]. Electrode referencing was done by bipolar subtraction using Data Science International implantable bipolar electrodes. The separation between the bipolar electrodes was of 5 mm (±1 mm). No further implanted reference or ground electrode was used to minimize the impact of the surgery on brain function.

After surgery, the lemurs returned to their individual cage. They were allowed to recover for 15 days from the beginning of the EEG experiments. The week after the surgery, nociception was minimized by subcutaneous daily injection of painkiller and anti-inflammatory drug (i.e. Metacam) under the control and supervision of an expert scientist and veterinarian (Dr. Fabienne Aujard, Ph.D.). Before the beginning of the experiment, animals were raised on fresh fruit and a mixture of cereals, milk and egg prepared daily in the breeding facility of UMR CNRS-MNHN 7179 (Brunoy, France). Water and food were given ad libitum.

Few days before the planned experiments, the candidate animals were provided with a wooden nest box and laurel branches for the enrichment of their environment in the individual cages above mentioned. As an important aspect of the experimental procedures, health status and general behavior of the candidate lemurs were examined to decide if they could be admitted to electrophysiological recordings after the surgery. The examination strictly followed the ARRIVE guidelines (i.e. “Animal Research: Reporting of In Vivo Experiments”; https://www.nc3rs.org.uk/arrive-guidelines). First, Dr. Fabienne Aujard carefully checked the complete healing of the surgical incisions. Second, the recovery of body temperature, body weight, and locomotor activity (recorded by automated telemetric system placed in the home cage of the animal) were verified in the 3 days preceding the electrophysiological experiments with respect to pre-surgery period. The animals were admitted to the electrophysiological recordings if they showed no loss of 2 g/day and no substantial decrease in locomotor activity (i.e. 50% or more) and temperature during these days. All animals were admitted to the electrophysiological recordings.

### Physiological data recording and analysis

During the present experiments, the lemurs were housed in their individual cages provided with branches and a wooden nest in which temperature and humidity were controlled and maintained constant (ambient temperature = 24–26°C, relative humidity = 55%). Food and water were available ad libitum (availability of food and water was kept constant according to the general conditions of captivity in the colony; [2]. Normal circadian rhythms were artificially set as follows: 14 h lights on and 10 h lights off.

Electrophysiological and behavioral data were acquired by telemetry during 5 consecutive days (Dataquest Lab Pro v. 3.0; Data Science International, St. Paul, MN, USA). The data for the present study were taken from the first experimental day.

EEG and EMG signals were acquired and exported by Neuroscore software v. 2.1 (Data Sciences International, St. Paul, MN, USA). As mentioned above, the EEG recording was performed from one EEG bipolar channel located in the premotor frontal cortex (500 Hz sampling rate; 1–100 Hz bandpass). The EMG signals were collected from one EMG channel using bipolar electrodes sutured to the neck muscles with non-absorbable polyamide suture (500 Hz sampling rate; 1–100 Hz bandpass). The temperature was also recorded (250 Hz sampling rate, range 34–41°C) using a thermistor integral to the electronics module inside the implanted transmitter. Locomotor activity was indexed by the variation of electromagnetic signal strength, recorded on a dedicated channel of the telemetry system (1 Hz sampling rate).

Electrophysiological recordings were performed for 1 wake-sleep cycle. As mentioned above, this cycle was divided into 14 h of daytime (It was the rest phase as lemurs are nocturnal animals) and 10 h of nighttime (The stage of most animal activities). Data analysis focused on a recording segment of 1 h during nighttime, namely the light-off period. It also focused on a recording segment of 1 h during the daytime, namely the light-on period. These 1-h recording segments were taken starting from 1 h after the light-off/on instant. This instant switched from daytime to nighttime and vice versa. The recording segments started 1 h after this instant to minimize the effects of stress due to the changes of environmental lightening. In these recording segments, behavioral and EEG data were analyzed to identify the first 2–5 min of artifact-free EEG data in the passive behavioral state and the first 2–5 min of artifact-free EEG data in the active behavioral state. The general procedure of this preliminary analysis is described in the following paragraphs.

Continuous EEG recordings were segmented into contiguous periods of 10 s for the analysis of animal behavior. Any period of 10 s was rated based on the prevalent behavior of the animal (“behavioral periods”). The classification of the behavioral periods was performed in two steps. In the first step, these periods were automatically classified by expert experimenters of Brunoy group (Dr. Anisur Rahman, Ph.D., or Dr. Fabien Pifferi, Ph.D.) using Neuroscore software v. 2.1 (Data Sciences International, St. Paul, MN, USA). This software classified the periods into mutually exclusive behavioral states such as active wake (i.e. active state) and passive wake (i.e. passive state) of interest for the present study and other states. The classification of Neuroscore software was based on the collected EEG, EMG, temperature, and locomotion variables (Brunoy group used this procedure successfully in previous studies [2, 34]). In the second step of the procedure, the results of such automatic classification were revised by Dr. Rahman and Dr. Pifferi, based on the visual inspection of the EMG activity, temperature, and locomotion data. They did not take into account the EEG data to avoid the risk of circular reasoning. Indeed, they could have unconsciously biased their judgments to confirm the experimental hypothesis of a reduction in amplitude of alpha rhythms during active compared to passive state. For example, they could have denoted as “awake passive state” the periods characterized by a high amplitude of on-going alpha rhythms. On the contrary, they could have denoted as “active passive state” the periods characterized by a low amplitude of on-going alpha rhythms. As a consequence, this circular reasoning would have determined the confirmation of the working hypothesis of the present study. The main scope of this action was to check that lemur was not sleeping in the periods classified as “passive state”, and was effectively active in the periods classified as “active state”. As a methodological remark, the experimenter always discarded periods framed in long time windows (minutes) of continuous permanence in the passive behavioral state (risk of drowsiness or initial sleep). Optimal periods of “passive state” were those in which the animal intermingled few periods of 10 s of passive state with periods of overt behavioral activity. The mean lengthiness of the data of continuous quiet wakefulness that were used to extract the behavioral periods of passive state was 25.1 s (±7.7 standard error, SE) in the daytime and 26.8 s (±6.2 s SE) in the nighttime. On average across the animal group, the amount of the behavioral periods of interest was 12.8 (±4.1 SE) for the passive state during the daytime, 13.8 (±6.3 SE) for the passive state during the nighttime, 71.9 (±18.6 SE) for the active state during the daytime, and 164.5 (±17.5 SE) for the active state during the nighttime. All lemurs showed sufficient periods of active state during the daytime and nighttime (i.e. an arbitrary amount of more than 10 periods). Furthermore, 11 out of 13 lemurs showed sufficient periods of the passive state during the daytime (i.e. all except that L#4 and L#10). Finally, 6 out of 13 lemurs showed sufficient periods of the passive state during the nighttime (i.e. discarded lemurs were named L#1, L#2, L#3, L#4, L#11, L#12, and L#13).

Any behavioral period of 10 s was segmented into 5 single contiguous epochs lasting 2 s for the preliminary analysis of artifacts in the EEG signal. Of note, the fragmentation of any behavioral period into contiguous EEG epochs of 2 s allowed minimizing the amount of EEG signals rejected for artifacts. Without this fragmentation, the presence of an artifact of 3–4 s in a single behavioral period of 10 s would have caused the rejection of a whole period of 10 s of EEG signal.

The analysis of artifacts in the EEG epochs showed the following findings at the group level. There was no EEG epoch with artifacts in the passive state during the daytime. Whereas, the percentage of EEG epochs with artifacts was 2.6% (±1.3% SE) for the passive state during the nighttime. Furthermore, the percentage of EEG epochs with artifacts was 1.1% (±0.7% SE) for the active state during the daytime and 7.3% (±3.1% SE) for the active state during the nighttime. Accordingly, for the passive state the amount of artifact-free EEG epochs was on average 75.5 (±22.3 SE) and 143.5 (±49.2 SE) during the daytime and nighttime, respectively. For the active state, the amount of artifact-free EEG epochs was on average 354.8 (±99.1 SE) and 769.0 (±83.5 SE) during the daytime and nighttime, respectively. Table 1 reports the mean (± SE) percentage and the amount of EEG epochs with and without artifacts for behavioral states and the times of interest. The EEG epochs with artifacts were rejected while those without artifacts were used for further data analysis.

**Table 1:** Mean (± standard error, SE) percentage and the amount of electroencephalographic (EEG) epochs with and without artifacts in the active and passive behavioral states. These values refer to the daytime and nighttime periods of interest.

	Daytime			Nighttime		
	Mean of artefact-free epochs (±SE)	Mean of rejected epochs (±SE)	Ratio of rejected/artefact-free epochs (%; ±SE)	Mean of artefact-free epochs (±SE)	Mean of rejected epochs (±SE)	Ratio of rejected/artefact-free epochs (%; ±SE)
**Active**	354.8 (±99.1)	4.5 (±3.6)	1.1% (±0.7%)	769.0 (±83.5)	53.6 (±21.2)	7.3% (±3.1%)
**Passive**	75.5 (±22.3)	0.0 (±0.0)	0.0% (±0.0%)	143.5 (±49.2)	6.5 (±3.4)	2.6% (±1.3%)

A control statistical analysis was performed to compare the amount of artifact-free EEG epochs between the passive and active states in the daytime and nighttime (Student t-test, p<0.05 two-tailed). The results showed a higher amount of artifact-free EEG epochs in the active state compared to the passive state in both daytimes (t = 2.99, p<0.01) and nighttime (t = 3.13, p<0.05).

EEG power spectrum analysis of the artifact-free EEG epochs was performed by a standard (Matlab; MathWorks, Natick, MA, USA) FFT algorithm using Welch technique and Hanning windowing function (no phase shift) with 1 Hz frequency resolution. A normalization of the EEG data was obtained by dividing the EEG power density at each frequency bin with respect to the EEG power density averaged across all frequencies from 0 to 100 Hz (excluding power density peak due to the utility frequency line frequency at 50 Hz). After such normalization, the solutions lost the original physical dimension. They were represented by an arbitrary unit scale in which "1" means absolute power density equal to the average of absolute EEG power density from 0 to 100 Hz while "2" means the double of that mean etc.

At this early stage of research, narrow EEG frequency bands with shared frequency bins were used to avoid any a priori hypothesis about EEG banding. These bands were the following: 1–2 Hz, 2–4 Hz, 4–6 Hz, 6–8 Hz, 8–10 Hz, 10–12 Hz, 12–20 Hz, and 20–30 Hz. Sharing of a frequency bin by two contiguous frequency bands has the theoretical advantage that one cannot assume that these frequency bands reflect distinct underlying neurophysiological mechanisms [26, 35, 36].

EMG data of artifact-free EEG epochs were rectified using a proprietary home-made routine written with MatLab programming language (MathWorks, Natick, MA, USA) [37]. The mean rectified EMG was used as an index of muscular activity for the correlation with spectral EEG markers.

### Analysis of lemurs' behavioral states

An important step of the data analysis was the classification of animal behavioral state during the EEG periods of interest (see previous section) in terms of active state vs. awake passive state. To this purpose, we used instrumental markers of movement and EMG activity. NeuroScore software (Data Science International, St. Paul, MN, USA) was used to classify periods lasting 10 s each into the following behavioral classes:

1. ACTIVE STATE. Overt exploratory movements.
2. AWAKE PASSIVE STATE. Immobility or small movements but absolutely no sleep. To be sure that the animal was not sleeping, experimenter classified as “awake passive state” a 10 s period in which the animal was mostly in a passive behavioral state and he/she was reasonably sure that the animal was not sleeping. To this aim, experimenter always discarded long periods (minutes) of behavioral passive state. And the preference was for periods in which the animal intermingled periods of 10 s of passive state with periods of overt behavioral activity.

### Statistical analysis

Statistical comparisons were performed in the following statistical sessions.

In the first statistical session, we tested the main hypothesis of a desynchronization of alpha rhythms (8–12 Hz) in the active state compared to the passive state (during both daytime and nighttime), as a translational oscillatory EEG marker of motor and locomotor activity. To this aim, two analyses of variance (ANOVAs) were performed, one for the daytime (N = 11 lemurs having sufficient EEG-EMG epochs in daytime and nighttime), the other for the nighttime (N = 6 lemurs having sufficient EEG-EMG epochs both in active and passive states). The normalized EEG power density was used as a dependent variable. The ANOVA factors were Condition (passive, active; independent variable) and Band (1–2 Hz, 2–4 Hz, 4–6 Hz, 6–8 Hz, 8–10 Hz, 10–12 Hz, 12–20 Hz, and 20–30 Hz). Mauchly's test evaluated the sphericity assumption when necessary. Correction of the degrees of freedom was made by Greenhouse–Geisser procedure. Duncan's test was used for post hoc comparisons (p<0.05 one-tailed). For each ANOVA, the working hypothesis would be confirmed by the following two statistical results: (i) an ANOVA interaction effect including the factor Condition (p<0.05); (ii) a post hoc test indicating that normalized EEG power density in the alpha range (8–12 Hz) was lower in active than in passive state (Duncan test, p<0.05 one-tailed).

As the number of EEG epochs was higher in the active compared to the passive state, a control analysis was performed to pair this variable. The number of EEG epochs in the active state was sub-sampled not to differ statistically from that of the passive state both in daytime (p>0.8) and nighttime data (p>0.2). Two ANOVAs were performed with the same features described in the previous paragraph.

In the main statistical analysis, contiguous EEG frequency bands shared a frequency bin. This methodological option was based on the assumption that contiguous frequency bands might not reflect distinct underlying neurophysiological mechanisms. However, it may artificially correlate the levels of that factor. Therefore, a control analysis was performed to test if the main results were confirmed even with non-overlapping contiguous EEG frequency bands. Specifically, two ANOVAs were performed. One ANOVA used the daytime data (N = 11 lemurs having sufficient EEG-EMG epochs both in active and passive states) while the other ANOVA used the nighttime data (N = 6 lemurs having sufficient EEG-EMG epochs both in active and passive states). In the two ANOVAs, the normalized EEG power density was used as a dependent variable. The ANOVA factors were Condition (passive, active; independent variable) and Band (1–2 Hz, 3–4 Hz, 5–6 Hz, 7–8 Hz, 9–10 Hz, 11–12 Hz, 13–20 Hz, and 21–30 Hz).

In the second statistical session, we tested the main hypothesis of a correlation between the normalized EEG power density and rectified EMG activity in the active state (Pearson test, p<0.05). This correlation analysis was performed both in the daytime (N = 13 lemurs having sufficient EEG-EMG epochs in nighttime) and the nighttime data (N = 13 lemurs having sufficient EEG-EMG epochs in daytime). The frequency bands were those showing statistically significant effects of the above ANOVAs.

In the third statistical session, we tested the control hypotheses of a stability of the normalized EEG power density and the rectified EMG activity in the active (passive) state between daytime and nighttime. To this aim, two ANOVAs on the EEG dependent variable were performed. One ANOVA was computed for the active state (N = 13 lemurs having sufficient EEG-EMG epochs in daytime and nighttime), the other for the passive state (N = 5 lemurs having sufficient EEG-EMG epochs in daytime and nighttime). The ANOVA factors were Time (daytime, nighttime; independent variable) and Band (1–2 Hz, 2–4 Hz, 4–6 Hz, 6–8 Hz, 8–10 Hz, 10–12 Hz, 12–20 Hz, and 20–30 Hz). Mauchly's test evaluated the sphericity assumption when necessary. Correction of the degrees of freedom was made by Greenhouse–Geisser procedure. Duncan's test was used for post hoc comparisons (p<0.05 one-tailed). For each ANOVA, the control hypothesis would be confirmed by the no statistical effect showing a difference of EEG power density between daytime and nighttime (Duncan test, p>0.05 one-tailed). Concerning the rectified EMG activity, two Student t-tests were performed between daytime and nighttime (p<0.05 one-tailed). One was computed for the active state, the other for the passive state.

In the fourth statistical session, we tested the control hypothesis that the rectified EMG activity was greater in the active compared to the passive state (Student t-test, p<0.05 one-tailed). This comparison was performed both in the nighttime (N = 6 lemurs having sufficient EEG-EMG epochs in nighttime) and the daytime data (N = 11 lemurs having sufficient EEG-EMG epochs in daytime).

## Results

### Results of the first statistical session

**Fig 1:**
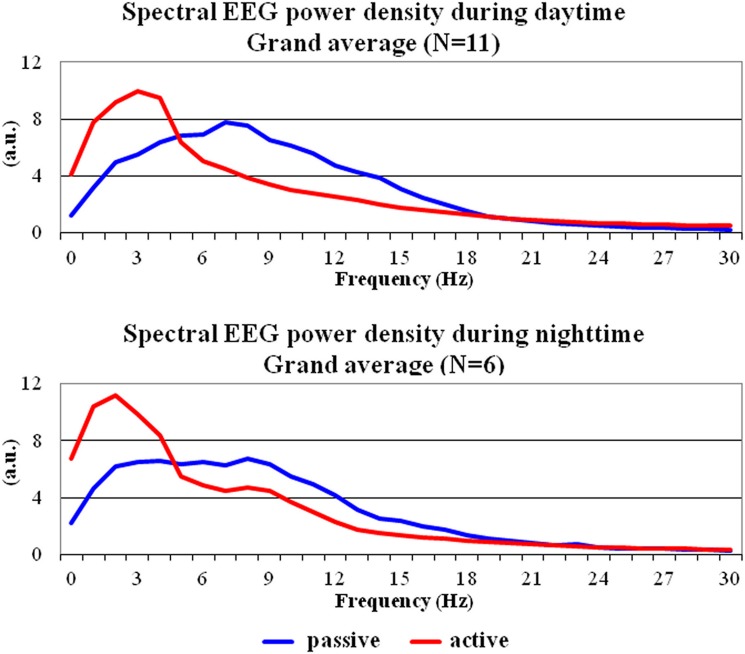
Grand-average across gray mouse lemurs of the normalized electroencephalographic (EEG) power density spectra between 0 and 30 Hz relative to the active and passive behavioral states. These spectra refer to the daytime (N = 11) and nighttime (N = 6) periods of interest. Lemurs having an insufficient amount of artefact-free EEG epochs in the passive state for the final analysis were not considered (daytime: L#4 and L#10; nighttime: L#1, L#2, L#3, L#4, L#11, L#12, and L#13).

Fig 1 shows the grand average across animals of the normalized EEG power density spectra (0–30 Hz) computed from lemur premotor cortex in the behavioral active and passive states. The data refer to daytime (N = 11 mouse lemurs having sufficient EEG epochs in both states) and nighttime (N = 6 mouse lemurs having sufficient EEG epochs in both states). In the EEG power density spectrum of the passive behavioral state, normalized EEG power density values showed a clear peak between 7 and 9 Hz, namely in the putative alpha range. Noteworthy, the normalized EEG power density values of this peak were lower (i.e. desynchronization) in the behavioral active compared to the passive state. Also, the normalized EEG power density values between 1 and 4 Hz, namely in the putative delta range, were higher (i.e. synchronization) in the former compared to the latter. This difference of the EEG variable was observed both in the daytime and nighttime data.

**Fig 2:**
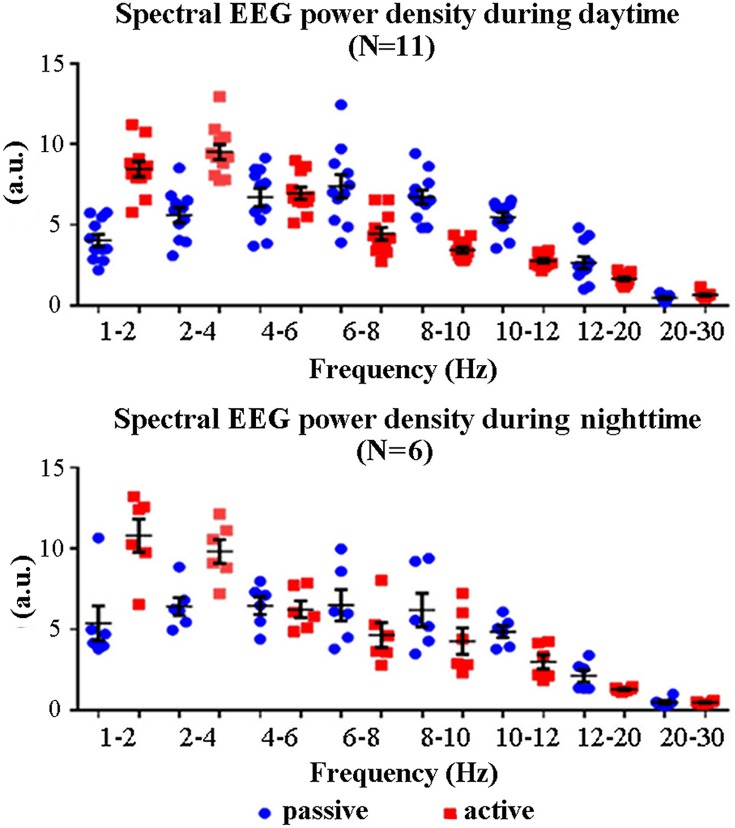
Individual EEG spectral power density values at frequency bands of interest (1–2 Hz, 2–4 Hz, 4–6 Hz, 6–8 Hz, 8–10 Hz, 10–12 Hz, 12–20 Hz, and 20–30 Hz) for any lemur (one circle = one lemur) and the active and passive states. These values refer to the daytime (N = 11) and nighttime (N = 6) periods of interest. Lemurs having an insufficient amount of artefact-free EEG epochs in the passive state for the final analysis were not considered (daytime: L#4 and L#10; nighttime: L#1, L#2, L#3, L#4, L#11, L#12, and L#13). The mean (± standard error, SE) EEG power density values are also plotted.

Fig 2 illustrates individual normalized spectral EEG power density values at frequency bands of interest (1–2 Hz, 2–4 Hz, 4–6 Hz, 6–8 Hz, 8–10 Hz, 10–12 Hz, 12–20 Hz, and 20–30 Hz) from premotor cortex and for all lemurs, states (active, passive), and times (daytime, nighttime). These values (circles) were relatively grouped with no substantial outlier.

**Fig 3:**
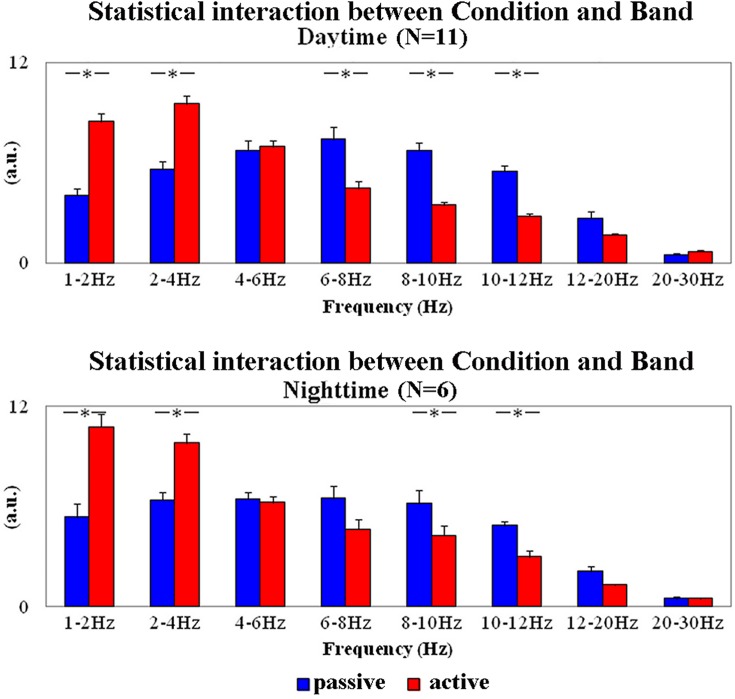
Mean values (± SE) of the normalized EEG power density in the active and passive states at the frequency bands of interest. These values refer to the daytime (N = 11) and nighttime (N = 6) periods of interest. Lemurs having an insufficient amount of artefact-free EEG epochs in the passive state for the final analysis were not considered (daytime: L#4 and L#10; nighttime: L#1, L#2, L#3, L#4, L#11, L#12, and L#13). In the figure, the illustrated values refer to the results of two ANOVAs. The first ANOVA showed a statistically significant interaction (F(7,70) = 34.556; p = 0.0001) between the factors Condition (active and passive states; independent variable) and Band (1–2 Hz, 2–4 Hz, 4–6 Hz, 6–8 Hz, 8–10 Hz, 10–12 Hz, 12–20 Hz, and 20–30 Hz) in the daytime. The second ANOVA showed a statistically significant interaction (F(7,35) = 16.103; p = 0.00001) between the same factors in the nighttime. Asterisks indicate the statistically significant differences (Duncan’s post hoc test; p<0.05).

Fig 3 plots the grand average across lemurs of the normalized EEG power density values at frequency bands of interest (1–2 Hz, 2–4 Hz, 4–6 Hz, 6–8 Hz, 8–10 Hz, 10–12 Hz, 12–20 Hz, and 20–30 Hz) in the daytime and nighttime data. These values refer to the main results of two ANOVAs aimed at testing EEG changes as a function of the behavioral states. The results of the first ANOVA showed an interaction between the factors Condition (active, passive; dependent variable) and Band (1–2 Hz, 2–4 Hz, 4–6 Hz, 6–8 Hz, 8–10 Hz, 10–12 Hz, 12–20 Hz, and 20–30 Hz) in the daytime data (F(7,70) = 34.556; p = 0.0001). The results of the second ANOVA showed the same interaction in the nighttime data (F(7,35) = 16.103; p = 0.00001). Duncan planned post hoc tests showed that in the daytime data, normalized EEG power density values were lower at 6–8 Hz, 8–10 Hz, and 10–12 Hz in the active compared to the passive behavioral state (p<0.00003). Furthermore, these values were higher at 1–2 and 2–4 Hz in the former compared to the latter state (p<0.00002). In the nighttime data, normalized EEG power density values were lower at 8–10 and 10–12 Hz in the active compared to the passive behavioral state (p<0.02). Furthermore, these values were higher at 1–2 and 2–4 Hz in the former compared to the latter state (p<0.00008).

For the first control purposes, the number of EEG epochs in the active state was sub-sampled to pair that of the passive state both in daytime (p>0.8) and nighttime data (p>0.2). Two ANOVAs were performed with the same features described in the previous paragraph. The results were quite similar to those of the main analysis. The first ANOVA showed a statistically significant interaction (F(7,70) = 42.287; p = 0.00001) between the factors Condition (active, passive; independent variable) and Band (1–2 Hz, 2–4 Hz, 4–6 Hz, 6–8 Hz, 8–10 Hz, 10–12 Hz, 12–20 Hz, and 20–30 Hz) in the daytime data. Duncan planned post hoc tests showed that normalized EEG power density values were lower in 6–8, 8–10, and 10–12 Hz in the active compared to the passive behavioral state (p<0.00002). Furthermore, these values were higher in 1–2 and 2–4 Hz in the former compared to the latter state (p<0.00003). The second ANOVA showed the same statistically significant interaction (F(7,35) = 14.885; p = 0.00001) in the nighttime data. Duncan planned post hoc tests showed that normalized EEG power density values were lower in 6–8, 8–10, and 10–12 Hz in the active compared to the passive behavioral state (p<0.03). Furthermore, these values were higher in 1–2 and 2–4 Hz in former compared to latter state (p<0.0001).

For the second control purpose, we tested if the main results were confirmed even with non-overlapping contiguous EEG frequency bands. The results were as follows. The first ANOVA showed a statistically significant interaction (F(7, 70) = 26.975, p = 0.00001) between the factors Condition (active, passive; independent variable) and Band (1–2 Hz, 3–4 Hz, 5–6 Hz, 7–8 Hz, 9–10 Hz, 11–12 Hz, 13–20 Hz, and 21–30 Hz) in the daytime data. Duncan planned post hoc tests exhibited that the normalized EEG power density values were lower at 7–8, 9–10, and 11–12 Hz in the active compared to the passive behavioral state (p<0.0001). Furthermore, these values were higher at 1–2 and 3–4 Hz in the former compared to the latter state (p<0.00003). The second ANOVA showed the same statistically significant interaction between the factors Condition and Band (F(7, 35) = 13.174, p = 0.00001) in the nighttime data. Duncan planned post hoc tests exhibited that the normalized EEG power density values were lower at 7–8, 9–10, and 11–12 Hz in the active compared to the passive behavioral state (p<0.03). Furthermore, these values were higher at 1–2 and 3–4 Hz in the former compared to the latter state (p<0.0001). The results of this control analysis confirmed those of the main analysis with overlapping EEG frequency bands.

The results of this statistical session unveiled reliable changes of delta and alpha rhythms in lemurs as a function of behavioral active and passive states.

### Results of the second statistical session

**Fig 4:**
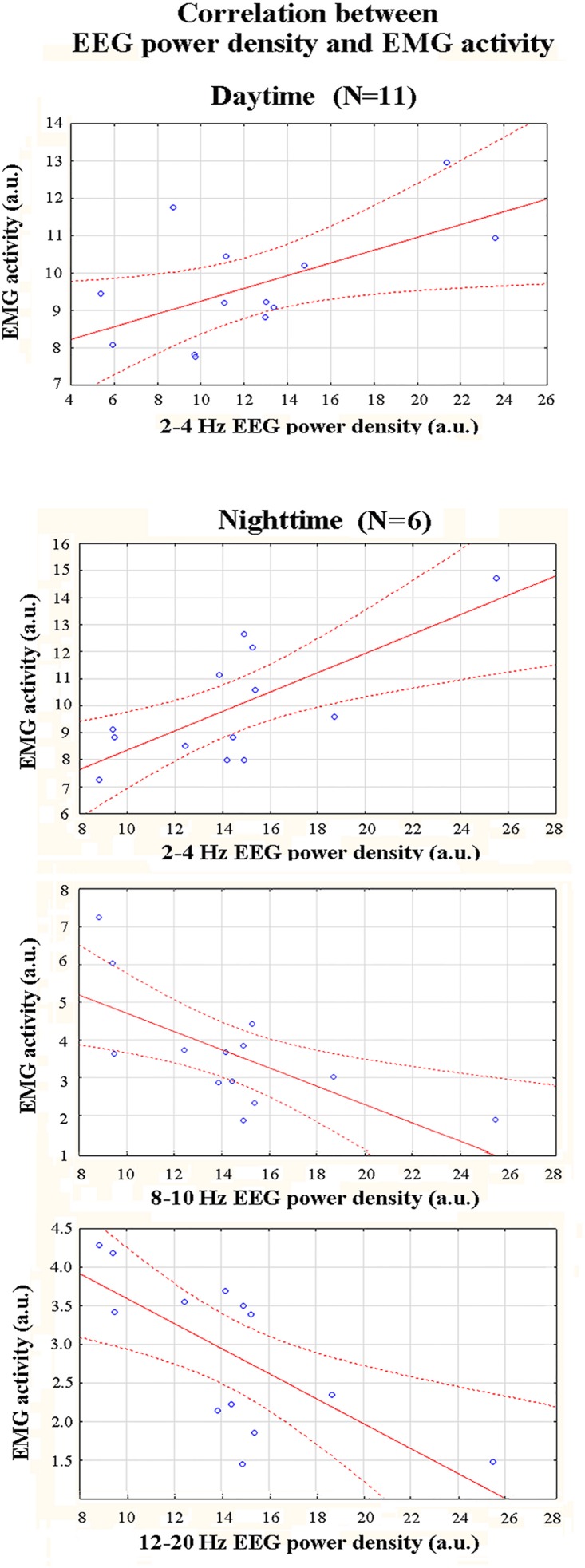
Scatterplots relative to the results of a correlation analysis (Pearson test; p<0.05) across gray mouse lemurs between the normalized EEG power density and the mean rectified EMG activity in the active state. These values refer to the daytime (N = 13) and nighttime (N = 13) periods of interest.

In this statistical session, a correlation analysis tested the relationship between the EEG and EMG variables in the active state. The frequency bands were those showing significant differences in the first statistical session (p<0.05). There was a statistically significant correlation between normalized 2–4 Hz EEG power density and the rectified EMG activity both in the daytime data (r = 0.58; p<0.03) and the nighttime data (r = 0.72; p<0.006). The higher the 2–4 Hz power density, the higher the rectified EMG activity. In the nighttime data, the correlation was negative between 8–10 and 10–12 Hz power density and the rectified EMG activity (r = -0.68; p<0.01 and r = -0.7; p<0.007, respectively). The lower this EEG power density, the higher the EMG activity. Fig 4 plots the results mentioned above.

The results of this session showed a robust correlation between the normalized EEG power density and rectified EMG activity in behaving lemurs, especially in the delta range.

### Results of the third statistical session

In this statistical session, two ANOVAs tested the stability of the EEG variables between daytime and nighttime. The first ANOVA on the active state data showed an interaction between the factors Time (daytime, nighttime; independent variable) and Band (1–2 Hz, 2–4 Hz, 4–6 Hz, 6–8 Hz, 8–10 Hz, 10–12 Hz, 12–20 Hz, and 20–30 Hz) (F(7,84) = 2.9455; p<0.009). Duncan planned post hoc tests showed that the only statistical EEG differences between daytime and nighttime were limited to 1–2 Hz (higher normalized EEG power density in the active compared to the passive state; p<0.0001).

The second ANOVA on the passive state data showed no statistically significant result (p>0.05).

In this statistical session, two Student t-tests evaluated the stability of the rectified EMG activity between daytime and nighttime. These tests showed statistical differences neither in the former nor the latter (p>0.05).

The results of this session showed a global stability of the present EEG (>2 Hz power density) and EMG variables in lemurs.

### Results of the fourth statistical session

**Fig 5:**
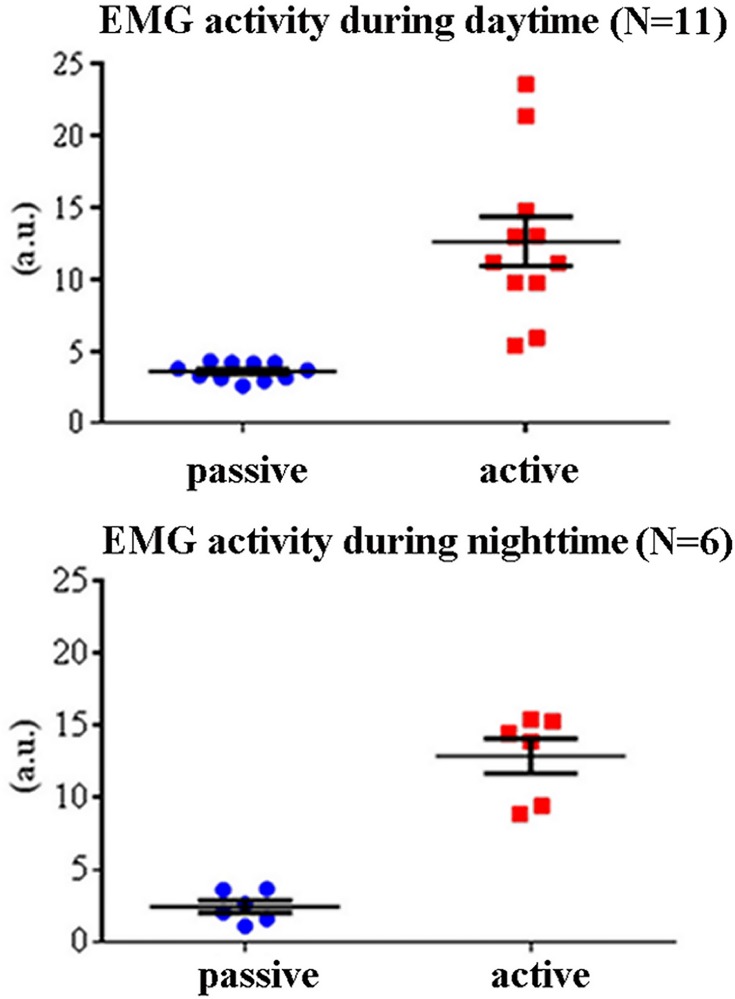
Individual values of the mean rectified EMG activity for any lemur and the active and passive states. These values refer to the daytime (N = 11) and nighttime (N = 6) periods of interest. The mean (± SE) of the rectified EMG activities are also plotted. Lemurs having an insufficient amount of artefact-free EEG epochs in the passive state for the final analysis were not considered (daytime: L#4 and L#10; nighttime: L#1, L#2, L#3, L#4, L#11, L#12, and L#13).

In this statistical session, two Student t-tests evaluated the control hypothesis that muscle activity was higher in the active compared to the passive state in both the daytime and nighttime. As expected, the results showed that rectified EMG activity was greater in the active compared to the passive state in both the daytime (t = 5.28, p<0.0003) and nighttime data (t = 10.01, p<0.0002). The results of this session confirmed the control hypothesis. Fig 5 plots the results mentioned above.

## Discussion

In humans, resting state EEG rhythms reflect the fluctuation of cortical arousal and vigilance in a typical clinical recording setting, namely the EEG recordings for few minutes in subjects placed in the state of eyes closed (i.e. passive condition) and eyes open (i.e. active condition). The higher the cortical EEG power at a given frequency, the higher the synchronization of cortical pyramidal neurons at that frequency [26]. Can this basic procedure be back translated to Gray mouse lemur (Microcebus murinus), which is considered a very useful primate model for translational research on aging [4, 5]? Typical methodology for preclinical EEG recordings in lemurs is based on the measurement of electrophysiological signals for tens of hours to extract markers of wake-sleep cycle and time course of EEG power in selected frequency bands over long periods [2, 34]. This time scale is quite different with respect to that of the typical clinical setting of EEG recordings in humans (i.e. tens of minutes). Therefore, EEG experts of the IMI PharmaCog project (www.pharmacog.org) planned this study aimed at testing a new procedure that mimics that of the mentioned clinical setting for humans. We compared on-going EEG rhythms between passive (i.e. quiet wakefulness with immobility or small movements of trunk, head, and forelimbs) and active (i.e. dynamic exploration of the cage) conditions recorded for a relative brief period of tens of minutes.

As novel findings, we report that lemurs showed ample alpha rhythms (peak at 7–9 Hz) in the premotor frontal cortex during the passive behavioral state defined in the methodology (i.e. quiet wakefulness). Compared with this passive state, an active behavioral state (i.e. motor and locomotor activity associated with exploratory movements) induced a desynchronization of alpha rhythms and the increase in the amplitude of delta rhythms (1–4 Hz). These findings were reliably observed in both daytime, which is the period of rest (lemurs are nocturnal), and nighttime, which is the period of most animal activities. Also, the enhancement of delta rhythms with motor-locomotor activity was reliably correlated with EMG activity in both daytime and nighttime. A tentative explanation of these findings can refer to the mechanism of generation of sensorimotor "mu" rhythms in the Rolandic cortex. Mu rhythms are constituted by two EEG oscillatory components. One component shows a frequency peak around 10 Hz (i.e. alpha rhythms) while the other peaks around 20 Hz (i.e. beta rhythms) [27, 28, 29]. In humans, Rolandic mu rhythms can be recorded by EEG, magnetoencephalography (MEG) and electrocorticography (ECoG). These rhythms are characterized by high amplitude (i.e. synchronization) in the condition of muscle relaxation (no muscle tension or movement), whereas they are blocked and disappear (i.e. desynchronization) during passive and active movements or isometric muscle tension [26, 28, 29, 35, 36, 37]. Pre-surgical subdural ECoG recordings in epilepsy patients unveiled fine spatial details of mu rhythm showing that movement-related alpha and beta desynchronization is localized in premotor, primary motor and somatosensory areas [29, 38].

Keeping in mind the above neurophysiological premises, a tentative explanation for the present findings is that humans, monkey and prosimian lemurs may share similar neurophysiological oscillatory mechanisms generating mu rhythms in the premotor frontal cortex. This similarity would represent an interesting translational model of synchronization at alpha frequencies (i.e. about 10 Hz) of premotor frontal neural populations in awake passive state and their desynchronization during preparation, execution, and control of biologically relevant voluntary movements. As an original theoretical contribution, the present explanation extends to prosimian mouse lemurs some neurophysiological features of alpha component of mu rhythms in premotor cortex previously shown in humans and monkeys [29, 30, 39].

Another interesting novel contribution of the present study is that in lemurs, premotor cortex showed neither a clear peak of beta power density around 20 Hz during passive behavioral state nor its reduction in amplitude during active behavioral state. This difference with respect to human frontal mu rhythms might be due to (1) a simpler functional organization of premotor frontal cortex in lemurs compared to humans; (2) an insufficient spatial sampling of frontal cortex in the present experiments (indeed, we recorded EEG activity only in premotor cortex with no sampling from primary motor and somatosensory areas). Future studies should clarify this issue by simultaneous EEG recordings from premotor, primary motor, primary somatosensory and superior parietal lobe areas.

As a further interesting finding of the present study, lemurs showed enhanced frontal delta rhythms (1–4 Hz) in the active compared with the passive behavioral state. Unfortunately, the current methodological approach did not allow disentangling the cognitive, motor, and locomotor processes underlying this modulation of delta rhythms. Concerning the cognitive counterpart, previous studies have shown that event-related delta rhythms are related to attention, stimulus encoding, episodic memory and decision-making processes in healthy subjects [40, 41]. Concerning the motor counterpart, enhancement of frontal event-related delta rhythms has been shown in healthy subjects during locomotion (i.e. treadmill walking) [42]. Interesting results on event-related delta rhythms have been also reported in pathological aging. It has been reported an abnormal amplitude of cognitive event-related delta rhythms in patients with mild cognitive impairment [43, 44] and AD [45, 46]. Furthermore, reactivity of frontal delta rhythms to eyes opening was lower in these patients with respect to normal elderly subjects [47, 48]. Keeping in mind these data, the present findings suggest that delta rhythms would represent another interesting translational neurophysiological model of cortical arousal for preclinical research in lemurs.

As a methodological limitation, the present EEG approach did not allow the investigation of the enhancement of cortical gamma rhythms (>40 Hz), ubiquitous in the brain [41, 49], and typically related to cognitive-motor processes in humans [26, 50]. The investigation of these rhythms would have required alignment of the onset of discrete motor events EEG epoch-by-epoch, to capture the event- and phase-locked gamma responses. Indeed, the present EEG approach was designed to study on-going premotor rhythms related to continuous motor and locomotor activity in freely behaving lemurs, as a back translation of the typical clinical setting of EEG recordings in humans (i.e. resting state eyes closed and open).

In conclusion, we tested the hypothesis that prosimian gray mouse lemurs showed frontal alpha rhythms in awake passive state and their desynchronization during motor and locomotor activity, as a promising translational neurophysiological model. Results showed ample alpha rhythms (peak at 7–9 Hz) in the premotor frontal cortex during the passive behavioral state. During the active state, these rhythms desynchronized in association with enhancement of frontal delta rhythms (1–4 Hz). These alpha and (especially) delta rhythms were correlated with EMG activity in the active behavioral state. The present results suggest for the first time that prosimian lemurs and humans partially share basic neurophysiological mechanisms of generation and modulation of frontal delta and alpha rhythms in quiet wakefulness and exploratory activity. This EEG approach may be an ideal translational back translation of the typical clinical setting of EEG recording in humans (i.e. resting state eyes closed and open) for basic (motor science) and applied (drug discovery) research.
